# Laparoscopic Cholecystectomy for Ruptured Gallbladder Varices Diagnosed by Preoperative Endoscopic Ultrasonography: A Case Report

**DOI:** 10.70352/scrj.cr.25-0740

**Published:** 2026-03-19

**Authors:** Takahisa Hirano, Masayoshi Nishihara, Michitaka Honda, Shima Asano, Naoya Ozawa, Shoko Himeiwa, Hirokatsu Iida, Shiho Terada, Hiroaki Kawamitsu

**Affiliations:** 1 Department of Surgery, Okinawa Prefectural Miyako Hospital, Miyakojima, Okinawa, Japan; 2 Department of Surgery, Southern Tohoku General Hospital, Koriyama, Fukushima, Japan

**Keywords:** cholecystectomy, laparoscopic, endosonography, hemorrhage

## Abstract

**INTRODUCTION:**

Gallbladder varices (GBV) are a rare form of ectopic varices associated with portal hypertension. They are often difficult to diagnose prior to rupture, which carries a high mortality rate. We report a case of ruptured GBV successfully treated with laparoscopic cholecystectomy following a definitive preoperative diagnosis by endoscopic ultrasonography (EUS).

**CASE PRESENTATION:**

A 43-year-old male with a history of alcoholic cirrhosis presented with epigastric pain. Contrast-enhanced CT revealed massive ascites and extravasation from the gallbladder wall, indicating active intra-abdominal hemorrhage. However, the specific etiology remained unclear. Transabdominal ultrasonography was suboptimal due to massive ascites and intestinal gas. Given the patient’s high risk for emergency laparotomy in a resource-limited setting and stable hemodynamics, we opted for overnight conservative management. The following morning, EUS was performed to definitively identify the bleeding source and exclude other upper gastrointestinal bleeding. EUS revealed prominent blood flow around the gallbladder neck suggestive of varices. Based on these comprehensive findings, a diagnosis of ruptured GBV was established. This definitive diagnosis allowed us to select a minimally invasive laparoscopic cholecystectomy. The postoperative course was uneventful, and the patient was discharged on POD 7. Although strict follow-up was planned, the patient was lost to follow-up. He died 1 year later from another gastrointestinal hemorrhage.

**CONCLUSIONS:**

Blood flow evaluation with EUS is valuable for the definitive diagnosis of ruptured GBV, especially when other imaging modalities are inconclusive. Accurate preoperative diagnosis by EUS enables the selection of laparoscopic cholecystectomy as a safe and effective therapeutic option, avoiding high-risk emergency open surgery in patients with liver cirrhosis.

## Abbreviations

EUS
: endoscopic ultrasonography
GBV
: gallbladder varices

## INTRODUCTION

Ectopic varices are varices that develop outside the esophagus and stomach in patients with portal hypertension. They carry a fourfold higher risk of bleeding than esophageal varices and have a reported mortality rate of approximately 40%.^1)^ GBV, a type of ectopic varices, frequently remain undiagnosed until rupture. Furthermore, ruptured GBV is often fatal, even with therapeutic intervention. We herein report the case of a patient who presented with intra-abdominal hemorrhage due to ruptured gallbladder varices and was successfully treated with emergency laparoscopic cholecystectomy.

## CASE PRESENTATION

**Fig. 1:**
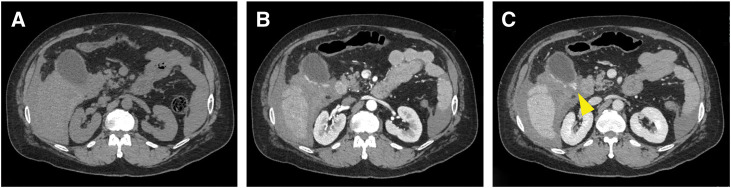
Dynamic CT findings. (**A**) Plain CT. (**B**) Arterial phase showing early enhancement on the dorsal aspect of the gallbladder. (**C**) Parenchymal phase showing extravasation from the same area (yellow arrow).

A 43-year-old male with a history of recurrent epigastric pain and heavy alcohol consumption presented with epigastric pain. A workup 1 year prior had revealed liver dysfunction. At presentation, the patient’s vital signs were as follows: blood pressure, 113/85 mmHg; heart rate, 75 beats/min; SpO_2_, 96% (room air); temperature, 37.1°C. The abdomen was soft and distended. Laboratory tests revealed an aspartate aminotransferase of 68 U/L, an alanine aminotransferase of 56 U/L, a total bilirubin of 1.4 mg/dL, a hemoglobin level of 13.0 g/dL, platelet count 187 × 10^4^ /μL, and normal coagulation studies. Abdominal ultrasonography showed increased hepatic echogenicity and a hypoechoic area suggestive of ascites. Abdominal contrast-enhanced CT with a slice thickness of 1.25 mm revealed ascites and a cirrhotic appearance of the liver without splenomegaly or obvious collateral circulation, including esophageal or gastric varices. The portal vein was patent without thrombosis. The gallbladder was enlarged. In the arterial phase, early enhancement was observed on the dorsal aspect of the gallbladder. In the parenchymal phase, extravasation was identified in the same area (**Fig. 1**). Abdominal paracentesis revealed hemorrhagic ascitic fluid. Transabdominal color Doppler ultrasonography was also performed, but detailed evaluation of blood flow around the gallbladder was difficult due to the effects of massive ascites and intestinal gas.

Although these findings were suspicious for a ruptured GBV, a definitive diagnosis was not established. In our remote island setting, blood products were critically limited: only 2 units of packed red blood cells and fresh frozen plasma were stocked for each blood type, and platelet concentrates were completely unavailable. Given this shortage and the patient’s background of alcoholic cirrhosis, performing an emergency laparotomy at night without a definitive diagnosis was considered to carry a high risk. Since the patient remained hemodynamically stable, we decided to manage him conservatively until the next morning. By the following morning, the patient’s vital signs remained stable, and the decrease in hemoglobin levels was gradual. Therefore, we decided to perform EUS to definitively identify the bleeding source and exclude other upper gastrointestinal bleeding sources.

**Fig. 2:**
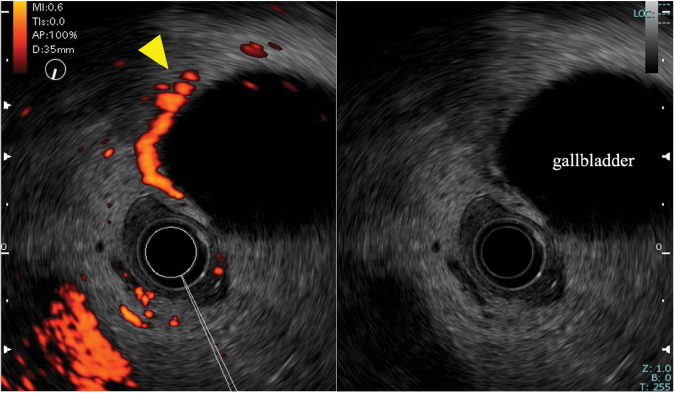
Endoscopic ultrasonography findings. Endoscopic ultrasonography revealed prominent blood flow around the gallbladder neck suggestive of varices (yellow arrow).

EUS revealed prominent blood flow around the gallbladder neck (**Fig. 2**). The observed flow pattern was continuous, a finding consistent with venous varices. Active extravasation could not be clearly identified. No esophageal or gastric varices, arterial aneurysms, common bile duct dilation, or other findings suggestive of hemobilia were observed. Therefore, based on the patient’s background of alcoholic cirrhosis, the CT findings of extravasation from the gallbladder, and the EUS findings suggestive of varices, a comprehensive diagnosis of ruptured GBV was made. Following EUS, while the patient’s vital signs remained stable, the hemoglobin level decreased to 9.6 g/dL. To ensure definitive hemostasis and a radical cure, we decided to proceed with emergency laparoscopic cholecystectomy.

**Fig. 3:**
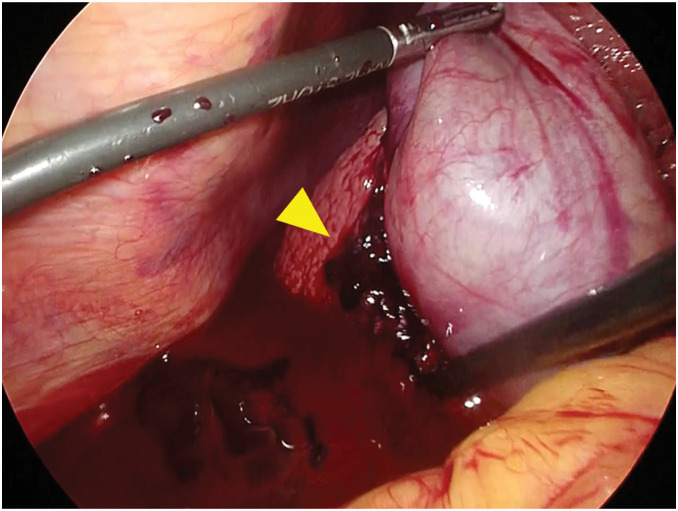
Intraoperative findings. Laparoscopic findings showed an adherent blood clot on the liver surface posterior to the gallbladder (yellow arrow).

**Fig. 4:**
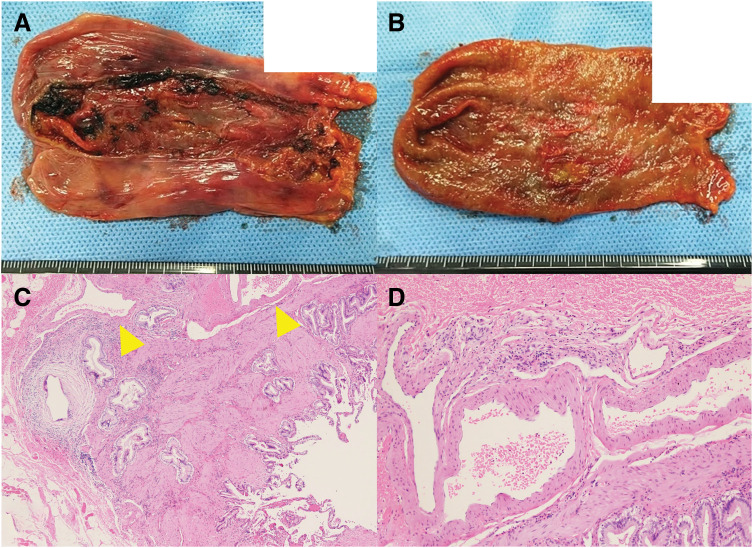
Pathological findings. (**A**, **B**) Serosal and mucosal surfaces of the resected gallbladder. (**C**) Histopathological finding showing dilated veins (yellow arrow) (hematoxylin and eosin stain, ×40). (**D**) High-power view of the varices (hematoxylin and eosin stain, ×100).

The surgical procedures were as follows (**Supplementary Video**). A five-port technique was used, consisting of the four conventional ports with an additional 5-mm assistant port placed to the right of the umbilicus. After bloody ascites was suctioned, laparoscopic findings revealed an adherent blood clot on the liver surface posterior to the gallbladder (**Fig. 3**). As no other obvious sources of bleeding were identified upon exploration, we proceeded with the planned laparoscopic cholecystectomy. During dissection of the gallbladder from the liver bed, there was venous bleeding from the dissection plane, which was controlled with electrocoagulation and clipping and the application of an absorbable hemostatic agent. After the abdominal cavity was irrigated, a drainage tube was placed on the dorsal side of the liver. The operative time was 115 minutes with an estimated blood loss of 2000 mL, requiring the intraoperative transfusion of 4 units of packed red blood cells and 4 units of fresh frozen plasma. Histopathological examination showed a normal mucosal surface, while irregularly thickened and dilated veins were observed in the submucosal and serosal layers, consistent with varices. No malignant findings were identified (**Fig. 4**).

The patient had an uneventful postoperative course, required no blood transfusions, and was discharged on POD 7. A post-discharge workup confirmed alcoholic cirrhosis. Although strict follow-up, including esophagogastroduodenoscopy to screen for other ectopic varices, was planned, the patient was lost to follow-up after discharge. Eight months after surgery, he was readmitted for esophageal variceal bleeding treated endoscopically. One year postoperatively, he died from a fatal gastrointestinal hemorrhage. Autopsy imaging confirmed the absence of intra-abdominal hemorrhage, supporting the diagnosis of death due to exsanguination from a gastrointestinal source.

## DISCUSSION

A rare case of ruptured GBV was successfully treated with laparoscopic cholecystectomy following a preoperative diagnosis by EUS. This case offers several important insights for the diagnosis and surgical treatment of GBV.

GBV have been reported in 20%–30% of patients with portal hypertension,^2,3)^ but the diagnosis is frequently missed prior to rupture. The reasons for this are twofold. First, they are often asymptomatic until rupture.^2,3)^ Second, their detection on CT or MRI is difficult due to their small caliber and abundant collateral pathways.^4)^ Our literature search using the keyword “gallbladder varices” identified 10 case reports on GBV, including the present case (**Table 1**). A definitive diagnosis was established clinically in five of these cases, and this included incidental findings in asymptomatic patients.

**Table 1:** Summary of Previous Reports

No.	Year	First author	Age (years)	Sex	Underlying disease	Symptom	Diagnosis	Treatment	Outcome (follow-up period)	Cause of death
Ruptured cases										
1	1991	Hellerich^6)^	50	Male	Alcoholic liver cirrhosis	Sudden death	Autopsy	None	Death (immediate)	GBV rupture
2	2002	Chu^2)^	41	Male	Alcoholic liver cirrhosis	Sudden death	Autopsy	Resuscitation	Death (immediate)	GBV rupture
3	2009	Kevans^3)^	43	Male	HCV-associated cirrhosis	Hemorrhagic shock	Autopsy	TIPS	Death (3 days post-TIPS)	GBV rupture
4	2016	Pravisani^5)^	50	Male	Alcoholic liver cirrhosis	Hemorrhagic shock	Intraoperative finfing	Open-C	Death (1 day post-op)	GBV rupture
5	2025	Present case	43	Male	Alcoholic liver cirrhosis	Epigastric pain	EUS & CT	Lap-C	Death (12 months post-op)	GI bleeding
Non-ruptured cases										
6	1991	Kitano^7)^	51	Male	Portal vein obstruction due to trauma	Chest discomfort	EUS	NR	NR	NA
7	2005	Mishin^8)^	51	Female	Post-splenectomy for hypersplenism	Asymptomatic	AUS	Conservative	Survive (3 years)	NA
8	2016	Gnerre^4)^	76	Male	HCV-associated cirrhosis	Asymptomatic	AUS & CT	Conservative	Survive (At report)	NA
9	2018	Mehl^9)^	17	Female	Post-gastric bypass for obesity	Abdominal pain	AUS & MRI	Conservative	Survive (NR)	NA
10	2023	Tong^10)^	37	Male	Cryptogenic portal hypertension	Epigastric pain	AUS & CT	Conservative	Survive (short-term)	NA

AUS, abdominal ultrasonography; EUS, endoscopic ultrasonography; GBV, gallbladder varices; GI, gastrointestinal; HCV, hepatitis C virus; Lap-C, laparoscopic cholecystectomy; NA, not applicable; NR, not reported; Open-C, open cholecystectomy; TIPS, transjugular intrahepatic portosystemic shunt

In our case, a definitive diagnosis was subsequently established by performing EUS, which demonstrated prominent abnormal blood flow. The patient’s clinical presentation was also nonspecific. While CT demonstrated intra-abdominal hemorrhage post-rupture, it was not conclusive for the diagnosis of GBV rupture. EUS did not detect active extravasation but identified varices at the bleeding site, which was essential for the diagnosis. This highlights the crucial role of blood flow assessment for diagnosis of GBV. In the previously reported cases (**Table 1**), ultrasonography was performed in all cases where a diagnosis was achieved before rupture. These findings support the recognition of color Doppler ultrasonography as the gold standard for diagnosing GBV.^4)^ Thus, if GBV are suspected, color Doppler ultrasonography should be performed.

There is no established treatment strategy for the rupture of GBV. Pravisani et al.^5)^ also reported a case in which a patient underwent an emergency open cholecystectomy and hemostasis but could not be saved. In that case, the patient was in hemorrhagic shock without a definitive preoperative diagnosis, which necessitated an emergent open approach. By contrast, the patient in our case was successfully treated with a laparoscopic cholecystectomy. Laparoscopic surgery is less invasive than open surgery and may therefore offer significant benefits for high-risk patients with liver cirrhosis. However, it also has disadvantages such as the difficulty of laparoscopic hemostasis and the risk of managing unexpected other ectopic varices. In this case, the patient's hemodynamic stability allowed sufficient time for detailed evaluation using EUS. Establishing a definitive preoperative diagnosis then enabled the selection of a minimally invasive procedure.

The prognosis for ruptured GBV is extremely poor. The reasons for this include the presence of underlying liver cirrhosis with impaired coagulation, and the tendency of ectopic varices to bleed profusely because of their rich blood supply. In the previously reported cases (**Table 1**), four of the five patients who presented with rupture died due to hemorrhagic shock, with our case the only one to have been successfully managed. In our case, the patient survived a ruptured GBV only to die from another complication of cirrhosis a year later, demonstrating that the long-term prognosis is determined by the underlying liver disease. Therefore, management of the cirrhosis itself and clear patient counseling on prognosis are crucial.

Finally, this case highlights the challenges of emergency care in remote island settings. The absence of on-site radiology specialists during nighttime hours contributed to delayed definitive diagnosis, and chronic shortages of blood products, medical resources, and healthcare personnel may hinder timely intervention in emergency cases. Rapid collaboration with radiology specialists and early consideration of interventional radiology (IVR) may help facilitate prompt diagnosis and treatment in similar situations.

## CONCLUSIONS

Blood flow evaluation with EUS is valuable for the diagnosis of GBV rupture. Based on an accurate preoperative diagnosis, laparoscopic cholecystectomy can be an effective therapeutic option for ruptured GBV.

## SUPPLEMENTARY MATERIAL

Supplementary video Laparoscopic cholecystectomy for ruptured gallbladder varices

## DECLARATIONS

### Availability of data and materials

Data sharing is not applicable to this article as no datasets were generated or analyzed during the current study.
